# Reduced pain perception in children and adolescents with ADHD is normalized by methylphenidate

**DOI:** 10.1186/s13034-016-0112-9

**Published:** 2016-07-22

**Authors:** Nicole Wolff, Katya Rubia, Hildtraud Knopf, Heike Hölling, Julia Martini, Stefan Ehrlich, Veit Roessner

**Affiliations:** Department of Child and Adolescent Psychiatry, Faculty of Medicine, TU Dresden, Fetscherstrasse 74, 01307 Dresden, Germany; Department of Child and Adolescent Psychiatry, Institute of Psychiatry, King’s College London, London, UK; Department of Epidemiology and Health Monitoring, Robert Koch Institute, Berlin, Germany

**Keywords:** ADHD, Methylphenidate, Pain, Opioid system, Dopamine

## Abstract

**Background:**

The present study examined pain perception in children and adolescents with ADHD and the interaction between pain perception and the administration of methylphenidate (MPH) in order to generate hypotheses for further research that will help to clarify the association between ADHD diagnosis, MPH treatment and pain perception.

**Methods:**

We included 260 children and adolescents of the “German Health Interview and Examination Survey for Children and Adolescents” (KiGGS) and analyzed parent’s assessments of children’s pain distribution and pain perception, as well as the influence of MPH administration on pain perception in affected children and adolescents.

**Results:**

Pain perception was associated with ADHD and MPH administration, indicating that children and adolescents suffering from ADHD without MPH treatment were reported to have lower pain perception compared to both, healthy controls (HC) and ADHD patients medicated with MPH.

**Conclusion:**

We suggest that reduced pain perception in children and adolescents with ADHD not medicated with MPH may lead to higher risk tolerance by misjudgments of dangerous situations, expanding the importance of MPH administration in affected children and adolescents.

## Background

Attention deficit hyperactivity disorder (ADHD) is characterized by the core symptoms of inattention, hyperactivity and impulsivity [1] and has a higher prevalence (OR = 4.80) among boys compared to girls [2, 3]. Neuropsychological dysfunctions in ADHD are a matter of ongoing debate, emphasizing deficits e.g. in response inhibition, vigilance, timing and working memory [4–6]. Neural pathways underlying these deficits point to deficits within frontal-subcortical catecholaminergic networks, involving dopaminergic and noradrenergic innervation [1, 5]. Hence, deficits in dopaminergic neurotransmission seem to be highly relevant for the neurobiology and therefore targets of medication of ADHD.

Low-dose psychostimulants, including methylphenidate (MPH) and amphetamines are the most widely used medications for ADHD [7]. MPH has been shown to substantially reduce core symptoms of inattention, hyperactivity and impulsivity in up to 70 % of affected children [8, 9]. However, there are also negative effects of MPH e.g. on sleep behavior, i.e. some authors found MPH treatment associated sleep-onset difficulties in patients with ADHD of all ages [10, 11]. Despite consistent evidence that low doses of MPH influence dopaminergic deficits in the brain [1, 12], neural mechanisms underlying its clinical action are not entirely understood at present [12].

Growing evidence suggests that dopamine is involved not only in ADHD core symptoms but also in other perceptive deficits, such as color perception [13], time perception [6, 14, 15] and pain perception [16]. Furthermore, the dopamine system is also closely interrelated with the opioid system, which plays a crucial role in pain perception as well as reward and motivation [17]. The opioid and dopamine systems interact closely in their mediation of reward and motivation, which have been shown to be abnormal in ADHD [18]. In addition, there is emerging evidence that the opioid system is associated with impulsiveness in animals and humans [19] and with the mechanism of action of stimulant medication [20, 21]. Given the close interaction between these two systems, evidence for associations between the opioid system and both ADHD and stimulant mechanism of action, raises the question whether pain perception that is mediated by the opioid system may also be altered in ADHD.

In fact, in daily clinical care altered pain perception, particularly in younger children with ADHD, has often been observed. For example, an association between growing pain and childhood restless leg syndrome was observed, and it was shown that this effect was more often observed in children with ADHD as compared to controls [22].

However, to the best of our knowledge, only two studies examined pain perception in children with ADHD [23, 24]. Scherder et al. [23] tested pain perception in 50 children and adolescents with ADHD, their unaffected siblings and HC [23], assessed through the children’s pain inventory. The study found no differences in pain perception in children and adolescents with and without ADHD. However, the 35 unaffected siblings of children and adolescents with ADHD reported reduced intensity and emotionality of past pain experiences compared to the unrelated HC [23]. The authors suggested that the long-term exposure of non-affected siblings to the “physical aggressiveness of their affected siblings” might have resulted in the observation of lower pain perception in the non-affected siblings [23]. Unfortunately, medication status of children and adolescents with ADHD during the period when pain experience was assessed was not considered which might have masked possible group differences in pain perception. In the second part of the study, children and adolescents with ADHD and their unaffected siblings gave blood for genetic analysis and were asked to assess the intensity and emotionality of perceived pain after the venipuncture. For this analysis, children and adolescents were requested to abstain from taking medication for at least 48 h. Children and adolescents with ADHD compared to non-affected siblings reported reduced pain perception. The second study, inducing experimentally pain in adolescents with ADHD [24] analyzed whether there is an association between subjective and physiological responses to pain and the presence of a comorbid conduct disorder (CD) in adolescents with ADHD. They analyzed adolescents with pure ADHD in comparison to adolescents with ADHD plus CD and measured pain perception through thermal heat on the skin of the palm of the hand. In addition, they collected the skin conductance level (SCL) and questionnaire reports on self-reported pain threshold and pain tolerance times. It was observed that although adolescents with ADHD plus CD vs adolescents with pure ADHD reported significantly increased pain threshold time and tolerance, the physiological response and SCL was similar in both groups. It was thus emphasized that it is important to consider comorbidities of ADHD when developing interventions. Moreover it was argued that it is important to bear in mind the interaction between aggression, antisocial behaviour, conduct disorder, and pain in the ADHD population [24].

Two further studies investigated pain perception in adults with ADHD, both finding enhanced pain [16, 25]. One study reported increased pain perception in a small sample of 25 adults with ADHD relative to 23 controls, assessed via a numerical pain rating scale, as well as more widespread pain, analyzed using a so-called pain drawing procedure [26]. Using a motor function neurological assessment (MFNU) in addition, they observed also that adults with ADHD compared to controls had motor inhibition problems and heightened muscle tone e.g. in the latissimus dorsi and calf muscles [25]. Pain location and pain levels were furthermore positively correlated with the total score on the MFNU, indicating that the pain reported in the ADHD group might be a consequence of their muscle tone dysregulation and motor inhibition problems [25]. However, most patients were responders to stimulant medication and the study did not state how many or whether any of the patients were medication-naive.

In the other study on pain perception in 30 adults with ADHD, pain was experimentally induced by 1 °C cold water. Adults with ADHD were more sensitive to pain [16] but pain perception was modified by MPH: adults with ADHD without MPH medication displayed lower pain threshold, i.e. a shorter interval from cold water exposure to the beginning of pain, and reduced pain tolerance, i.e. shorter interval participants can bear up against pain, in comparison to both, participants with ADHD medicated with MPH and HC.

In summary, pain perception seems to be altered in ADHD and to be influenced by the administration of MPH. Interestingly, although direct associations have not been investigated at this point, it can also be assumed that alterations in pain perception in ADHD may also be treated by the help of Neurofeedback [27, 28]. Here, frequencies in the alpha (about 10 Hz) and in the delta band (about 1–3 Hz) have been shown to influence muscle contraction as well as pain perception, which might also help to normalize pain perception in ADHD. Similarly, training of mindfulness has been associated with improvements in self-regulation of attention in ADHD [29] as well as with increased body perception. Thus a range of mind–body approaches may also be used in the management of altered pain perception [30]. All these treatments may thus be of benefit in the treatment of ADHD (both reduction of core symptoms and improvement of pain perception).

However, studies on pain perception have been inconclusive, with one study in children reporting no differences in past pain but reduced perception of induced pain between children with ADHD and HC [23], one study in adolescents comparing adolescents with pure ADHD and those with ADHD and comorbid CD reported similar physiological pain perception but decreased reported pain perception in adolescents with ADHD and CD as compared to adolescents with pure ADHD [24] and the two adult studies reporting enhanced pain perception [16, 25], which furthermore appeared to be modified/normalized by MPH [16]. The conflicting findings may be due to low power in small sample sizes or differential pain perception in different age groups of ADHD patients. However conflicting findings between studies could also be a result of differences in applied diagnostic criteria (DSM IV vs DSM 5 vs ICD 10). It has for example been observed that the manifestation of ADHD subtypes differs (i) between adolescents and adults and (ii) through the application of different diagnostic systems. Moreover, the diagnosis of ADHD as well as common comorbidities, for example autism spectrum disorders (ASD) differs depending on the diagnostic system used.

The purpose of the present study was to generate hypotheses for further research that will clarify the association between ADHD diagnosis, MPH treatment and pain perception. Thus we analyzed this research question in a large, representative sample of German children and adolescents. Although altered pain perception has been observed recurrently in children with ADHD in clinical praxis, to the best of our knowledge, no large-scale study has tested (1) pain distribution and pain perception in children with ADHD compared to that of HC and (2) the effect of MPH treatment on pain perception differences. Since alterations in pain perception, the mode of action of MPH and the neurochemical changes underlying ADHD are all linked to dopaminergic deficits in the brain, we hypothesized that pain perception would differ between children and adolescents with ADHD relative to HC and between medicated and non-medicated patients with MPH. Furthermore, given evidence that age and gender have an impact on pain perception [31–33], we also tested the effects of these two variables on potential group differences.

## Methods

The present study analyzed data from the German Health Interview and Examination Survey for Children and Adolescents called “KiGGS’’. KiGGS represents a nationwide, representative cross-sectional health interview and examination survey conducted in Germany from May 2003 until May 2006 by the Robert Koch Institute (RKI). The KiGGS study surveyed 17,641 children and adolescents aged from birth to 17 years from 176 cities and municipalities across Germany. The children and adolescents were physically examined and the father and/or mother (depending on which parent accompanied the child) as well as children over the age of 11 years completed a questionnaire covering psychological and social assessment. The study is fully compliant with the Declaration of Helsinki and was approved by the ethics committee at the University Hospital—Charité in Berlin and the Federal Office for the Protection of the Data. Signed informed consent was obtained from the primary caregivers of all study participants and also from all adolescents of 14 years or above. KiGGS consisted of a core survey (on which we access in the present study) and five additional subsample modules (on which we do not further refer here). Within KiGGS several self-administered questionnaires collecting data according to i.e. physical health, behavioral and emotional problems, social determinants of health, health-related behavior, health care service utilization and socio demographics were designed by the RKI. More details according to further objectives, design and measurements of KiGGS were reported elsewhere [34].

We focused on participants with available information regarding ADHD diagnosis, MPH medication, information about pain during the last 3 months, as well as “pain perception” (see the column “pain perception sample” in Table 1). Sixty-five participants met all these criteria (see participants of the category “ADHD with MPH” within the pain perception sample) and were compared to sixty-five unmedicated ADHD participants randomly selected from the remainder of the sample (n = 115). In addition, a similarly sized age-matched healthy control group without ADHD diagnosis and without any medication was randomly selected out of the remaining pain perception sample (n = 2687). In sum, the analyzed group (refer to the column with the heading “study sample” in Table 1) contains of 260 participants (50.0 % ADHD, 25.0 % MPH medicated, 70.8 % male) in the age range between 7 and 10 years (47.7 %), 11–13 years (31.5 %) and 14–17 years (21.2 %). Among those diagnosed with ADHD, 98 (75.4 %) were male and 32 (24.6 %) were female. For more details concerning the substitution of our sample, see Table 1.

**Table 1:** Sample description and statistics for participants’ age and gender

Whole KiGGS sample^a^ N = 13.488 (100 %)				Subsamples selected out of the whole KiGGS Sample by available information									
				MPH treatment sample^b^ N = 4637 (34.38 % of the whole KiGGS sample)			Pain perception sample^c^ N = 2867 (61.83 % of the whole KiGGS sample)			Study sample^d^ N = 260 (3.56 % of the whole KiGGS sample)			Participant Group N = 260 (100 %)
	HC N = 12828 (95.1 %)	ADHD sample N = 660 (4.9 %)	Whole *KiGGS* sample N = 13488 (100 %)	HC N = 4370 (94.2 %)	ADHD without MPH N = 182 (3.9 %)	ADHD with MPH N = 85 (1.9 %)	Whole sample N = 13488 (100 %)	ADHD without MPH N = 115 (4.01 %)	ADHD with MPH N = 65 (2.27 %)	HC N = 130 (50 %)	ADHD without MPH N = 65 (25 %)	ADHD with MPH N = 65 (25 %)	
*Participant age (years)*													
3–6	3509 (27.4 %)	57 (8.6 %)	3566 (26.4 %)	956 (21.9 %)	15 (8.2 %)	–	3566 (26.4 %)	6 (5.22 %)	–	–	–	–	
7–10	3542 (27.6 %)	215 (32.6 %)	3757 (27.9 %)	1255 (28.7 %)	58 (31.9 %)	37 (43.5 %)	3757 (27.9 %)	40 (34.78 %)	31 (47.69 %)	61 (49.6 %)	31 (47.7 %)	31 (47.7 %)	123 (47.3 %)
11–13	2581 (20.1 %)	202 (30.6 %)	2783 (20.6 %)	942 (21.6 %)	45 (24.7 %)	29 (34.1 %)	2783 (20.6 %)	27 (23.48 %)	19 (30.77 %)	41 (31.5 %)	22 (33.8 %)	19 (29.2 %)	82 (31.5 %)
14–17	3196 (24.9 %)	186 (28.2 %)	3382 (25.1 %)	1217 (27.8 %)	64 (35.2 %)	19 (22.4 %)	3382 (25.1 %)	42 (36.52 %)	15 (21.54 %)	28 (21.5 %)	12 (18.5 %)	15 (23.1 %)	55 (21.2 %)
Group difference	*χ*2 = 124.86, *df* = 3, *p* < 0.001			*χ*2 = 31.20, *df* = 3, *p* < 0.001			*χ*2 = 31.30, *df* = 3, *p* < 0.001			*χ*2 = 0.573, *df* = 4, *p* = 0.966			
*Gender*													
Male	527 (79.8 %)	6224 (48.5 %)	6751 (50.1 %)	71 (83.5 %)	143 (78.6 %)	71 (83.5 %)	6751 (50.1 %)	91 (79.13 %)	51 (78.5 %)	86 (66.2 %)	47 (72.3 %)	51 (78.5 %)	184 (70.8 %)
Female	6604 (51.5 %)	133 (20.2 %)	6737 (49.9 %)	14 (16.5 %)	39 (21.4 %)	14 (16.5 %)	6737 (49.9 %)	24 (20.87 %)	14 (21.5 %)	44 (33.8 %)	18 (27.7 %)	14 (21.5 %)	76 (29.2 %)
Group difference	*χ*2 = 246.45, *df* = 1, *p* < 0.001			*χ*2 = 38.07, *df* = 1, *p* < 0.001			*χ*2 = 23.63, *df* = 3, *p* < 0.001			*χ*2 = 3.27, *df* = 2, *p 0*.195			

*MPH* methylphenidate; *ADHD* attention deficit hyperactivity disorder

^a^Whole KiGGS Sample, represents the whole KiGGS sample (without missing data) regarding the classification ADHD diagnosis yes/no

^b^MPH Treatment Sample, includes only those participants out of the whole KiGGS Sample with available information regarding ADHD diagnosis and MPH medication

^c^Pain Perception Sample, includes only those participants out of the whole KiGGS Sample with available information regarding ADHD diagnosis, MPH treatment and information about pain perception

^d^Study Sample includes 65 participants, who confirmed to have ADHD, to take MPH and to have had pain during the last three months according to ratings in the Pain Perception Sample. In addition, an ADHD group (n = 65) without MPH medication and a HC group (n = 2 × 65 = 130) randomly have been selected out of the Pain Perception Sample to minimize/rule out that the homogeneity of variance assumption is affected. The three participant groups (ADHD with MPH, ADHD without MPH, HC) of the study sample do not differ with respect to age- or gender (all *p* > 0.195)

*ADHD diagnosis* Participants were allocated to the ADHD group, if they had ever been diagnosed with ADHD. For this purpose, parents were asked, whether their child was diagnosed with ADHD. If they answered “yes” it was further specified whether ADHD was diagnosed by a physician or psychologist. For an estimation of the validity of the parental answer, symptomatic information was correlated with the scale “hyperactivity” of the strengths and difficulties questionnaire (SDQ, Cronbach’s α = 0.78) [35]. In addition, a ROC analysis with the hyperactivity scale of the SDQ as explanatory variable and the diagnosis question as criterion was applied and revealed a AUC (region under the curve) of =0.86. [35]. Finally, although a clarified subtype specification, like in DSM IV [36, 37] into a predominantly hyperactive-impulsive type (ADHD-H), predominantly inattentive type (ADHD-I), and combined type (ADHD-C) took not place, parents of ADHD children were asked to specify whether ADHD or ADD was diagnosed.

*MPH medication* Data on the current intake of medication were collected by a standardized interview conducted by a physician. More information concerning applied methods, prevalence and determinants of ADHD medication can be found in Knopf [38] as well as Knopf et al. [3].

*Pain distribution and pain perception* To assess pain distribution and perception, parents as well as children and adolescents were asked about pain during the last 3 months. More specifically if children were in the age between 7 and 10 years parents were asked about their children’s pain, if children and adolescents were in the age range between 11 and 17 years they were asked directly about their own pain perception. If participants answered the question with “yes”, they were asked: “Which pain could be characterized as the “main pain”? The “main pain” (e.g. headache, back pain, ear pain, eye pain, stomach pain, arm or leg pain, toothache, etc.) needed to be specified according to “pain perception” (0 = hardly noticeable pain to 100 = maximum imaginable pain). For the purpose of this study we created a new variable “pain perception”, which contains both information the data from the parents (if children were younger than 11 years) as well as the data from children and adolescents their self (if children and adolescents were between 11 and 17 years). For more information on the measurement of pain via the KiGGS Data please refer to [39]. However, please note that the assessment of pain perception was measured via self-constructed questionnaires by the RKI, indicating that information according to validity or reliability of the measurement is missing.

*Statistical analyses* First, Chi square tests were conducted to analyze possible differences in sample characteristics (age and gender) between the three participant groups (ADHD with MPH, ADHD without MPH, HC). Second, Chi square tests were conducted to analyze the association between (a) “participant group” (ADHD with MPH, ADHD without MPH, HC) and the “type of main pain”, (b) “age” (7–10, 11–13, 14–17 years) and the “type of main pain” and (c) “gender” (male/female) and the “type of main pain”. Last, an analysis of covariance (ANCOVA) with the dependent variable “pain perception”, the independent variable “participant group” (ADHD with MPH, ADHD without MPH, HC) and the cofactors “age” (7–10, 11–13, 14–17 years) and “gender” (male, female) was conducted and Bonferroni corrected. All statistical analyses were conducted using SPSS 22 (SPSS, Inc., Chicago, Il, USA).

## Results

*Sample characteristics* (see Table 1). Chi Square test analyzing the association between “participant group” (ADHD with MPH, ADHD without MPH, HC) and “age” (7–10 years, 11–13 years, 14–17 years) in the study sample revealed no significant effect (χ2 = 0.573, df = 4, *p* = 0.966). In addition a similar analysis in the study sample between “participant group” and “gender” (female, male) revealed no significant effect (χ2 = 3.27, df = 2, *p* = 0.195). This showed no differences related to “age” or “gender” between the three participant groups, included in our study sample.

*Type of main pain* Chi Square test analyzed the association between “participant group”, “age”, “gender” and the “type of main pain”. This analysis revealed no significant effects (all *p* > 0.05), indicating that group reports of main pain type were independent of ADHD diagnosis, use of MPH medication, age or gender.

**Fig. 1:**
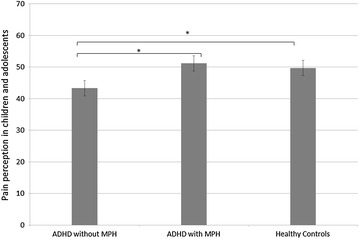
Differences in pain perception (0 = hardly noticeable pain to 100 = maximum imaginable pain) in children and adolescents with ADHD without MPH treatment (n = 65), children and adolescents with ADHD with MPH treatment (n = 65) and HC (n = 130). All children and adolescents were in the age range between 7 and 17 years. *Error bars* depict standard errors of the mean. The asterisk represents a significant difference on the p <.05 level

*Pain perception* (see Fig. 1). The analysis revealed a main effect for “participant group” on “pain perception” (*F*[2234] = 4.62, *p* = 0.011, *η*^*2*^ = 0.039). Post hoc t-tests indicate that children and adolescents with ADHD without MPH reported lower pain perception (*M* = 41.69, ± 21.27 *SD)* compared to HC (*M* = 50.2, ± 23.51 *SD; t*[193] = 2.46, *p* = 0.015) and compared to children and adolescents with ADHD and MPH (*M* = 51.17, ± 21.87 *SD; t*[128] = −2.51, *p* = 0.014). In addition, no significant differences in “pain perception” were observed between children and adolescents with ADHD and MPH and HC (*t*[193] = −0*.28, p* = 0.782). Since the variable “pain perception” represents a combined variable of analyzed data from children and adolescents (in the age between 11 and 17 years) as well as from parents (in the age between 7 and 10 years), we analyzed data separately as well. The analysis on parental (*F*[2152] = 4.35, *p* = 0.015, *η*^*2*^ = 0.055) as well as on children’s (*F*[2105] = 8.97, *p* = 0.003, *η*^*2*^ = 0.079) information revealed a significant main effect for “participant group” on “pain perception”. Both main effects report the same finding, as was observed in the analysis of the combined pain perception variable.

## Discussion

The present study examined alterations in pain perception and pain distribution in children and adolescents with ADHD relative to HC and the impact of MPH on these.

Pain distribution was similar among the three groups, indicating that no specific kind of somatic pain was predominant in any of the three participant groups. However, children and adolescents with ADHD without MPH reported lower pain perception compared to both, HC and stimulant-medicated children and adolescents with ADHD. Although the present study should be regarded as a first step, in which collected and analyzed data stem from questionnaires instead of a controlled experimental design, they offer a hint into the direction that MPH may enhance abnormally reduced pain perception in children and adolescents with ADHD up to the level of HC.

To our knowledge, this is the first large-numbered study on reduced pain perception in children with ADHD. The findings help to extend findings from a previous pilot study in children with ADHD that found reduced perception of induced pain [23] as well as findings of a study on pain perception in pure ADHD vs ADHD with comorbid CD [24]. However, they are not in line with the two studies testing induced pain perception via thresholds in adults with ADHD who found enhanced pain perception in controlled experimental settings [16, 25]. Furthermore, in the study of Treister et al. [16], MPH also seemed to normalize pain perception, however, in contrast to our study findings, by reducing the elevated pain perception up to the level of HC. These differences in findings between pediatric and adults studies could be explained by differences in the methodology of assessing pain perception, which in our study were assessed using parent-/child-rated questionnaires of their child’s/their own pain over the last 3 months while in the two adult studies they were tested experimentally by inducing pain [16, 25]. In fact, the mentioned pilot study in children [23] found no differences in questionnaire-assessed pain perception in children with ADHD but in the perception of induced pain, suggesting that the two methodologies assess different aspects of pain perception and are not directly comparable. In addition, external ratings (parent ratings) of the child’s pain may not be accurate given that pain is a subjectively felt perception and difficult to rate externally [40, 41]. However, our additional analysis, testing both responses (parents as well as children and adolescents) separately revealed identical effects. Still, measures of induced pain perception may potentially be more objective and sensitive than external ratings of someone else’s pain [42]. Accordingly the results of the present study should be regarded as a first step, although the results stem from a huge representative sample of German children and adolescents and could be used for generating more specific hypotheses for further research. In addition, recruitment is quite different between an epidemiological sample where parents mainly decide to participate and a smaller sample of clinically referred adults seeking help for their ADHD and participating in an experimental study. One possible explanation might be that those in the adult ADHD sample were generally less patient and more introspective than their HCs. If so, they might have been more impatient and less motivated to tolerate their experimentally induced pain, something that could have been improved by MPH. In addition, adults with ADHD have shown further characteristics, such as reduced educational performance, reduced attainment in workplace functioning as well as in the occupational attainment, increased risk for substance dependence and abuse, driving risks and irregularities in marital and interpersonal relations [43]. These specifics in combination with increased scores on avoidant, histrionic, narcissistic, negativistic, and self-defeating personality scales [44] may also explain the increased pain perception found in adults with ADHD. A difference between the existing studies that has been shown to impact on pain perception in general is age [45]. As in the rest of the population, adults with ADHD may have a different perception of pain than children with ADHD. Moreover, as stated in the introduction, differences in reported results of pain perception in ADHD may also rely on differences in applied diagnostic systems (IDC 10, DSM IV and DSM V) including the associated underestimation and partly non-consideration of comorbidities like ASD. Interestingly, in accordance to our findings on ADHD, an important feature of ASD is heightened or reduced sensitivity for pain [46, 47], pointing to differences between study results on pain perception whether participants with pure ADHD or with comorbid disorders like ASD [46, 47] or CD [24] were included in the sample.

Medication may potentially also be a confound, given that we found medication to upregulate reduced pain perception. Clinically recruited adults still suffering from ADHD are a specific subgroup of persisting ADHD and also have typically had a longer exposure to stimulant medication. This was in particular the case in the study of Stray [25] who recruited responders to stimulant medication only. It is possible that long-term stimulant medication over years in adults enhances the pain threshold in ADHD to the extent to elevate it to abnormally high levels.

The findings of reduced pain perception and its modulation by stimulant medication are also interesting in view of recent evidence that the opioid system is implicated in impulsiveness [19] and in the mechanism of action of stimulant medication [20, 21].

The opioid and dopamine systems interact very closely and are both crucially related to reward and motivation [17] which has been found to be abnormal in ADHD [18]. The opioid system has recently been found to be associated with impulsive behaviors such as inhibitory control and impulsive choice and higher opioid systems have been observed in healthy adults with impulsiveness traits [19]. In line with this, naltrexone, an opioid antagonist, improves self-control in impulsive disorders [48] and modulates brain regions implicated in motivational impulsiveness [49] that are typically abnormal in ADHD during reward-related processes such as ventral striatum and ventromedial orbitofrontal cortex [50]. An implication of the opioid system in impulsiveness hence may well also be associated with abnormal pain perception given the involvement of the opioid system in pain perception. The association with higher opioid levels in adults with impulsiveness behaviors [19] is in the same direction with our finding of reduced pain thresholds in children with ADHD. It can thus be speculated that reduced pain perception in unmedicated children with ADHD may be due to abnormally high opioid levels. However, in regard to the applied methods of our study such considerations should be taken with care, and need further experimental examination.

Furthermore, dopamine agonists such as amphetamines have been shown to release opioids in positron emission tomography studies in healthy adults [20, 21]. The findings suggest that stimulants do not work exclusively via the catecholamine systems but also via the opioid system. It also implies that the opioid system may be abnormal in ADHD, given the effectiveness of stimulants on ADHD behaviors. If opioids are released after stimulant medication in ADHD, like in healthy adults, then this could also explain the underlying mechanism of action of the modulation of pain perception in medicated ADHD children relative to unmedicated ones. In fact, stimulants enhance dopamine release in ADHD patients more than in healthy subjects [51], which would synergistically also enhance opioid levels, even if it was a purely downstream effect, which could then modulate the abnormally reduced pain perception. Considering these effects of psychostimulants on the opioid system, it is conceivable that long-term stimulant medication alters pain perception in ADHD subjects, which may well be different in adults who have been treated for longer time periods with psychostimulant medication than in children where stimulant medication periods are typically shorter.

If the finding, based on the preliminary character of the present study, of normalization of reduced pain perception by MPH treatment in children can be replicated in further studies, it would have clinical/public health as well as scientific implications. It offers an important link to the observation that MPH treatment is associated with a reduced risk of injury-related emergency department admission in children and adolescents with ADHD [52]. It could be argued that their reduced pain perception as well as poor concentration and increased impulsivity may underlie the increased risk of injury-related emergency department admission.

We suggest that in addition to cognitive factors, including lower expectations of personal risk in hazardous situations and less ability to generate prevention strategies and safety rules [53], reduced pain perception in children and adolescents with ADHD may also lead to higher risk tolerance by unfavorable effects on implicit learning as well as by misjudgments of dangerous situations. In view of the elevated rate of accident proneness [52, 54], a focus of ADHD behavioral treatment programs on both, better concentration abilities and reduced impulsivity, but also on psychoeducation in terms of decreased pain perception might possibly help to minimize one of the negative consequences of ADHD, as for example the greater distress, the time in treatment and the caused expenses, in those children not treated with MPH. In addition, the positive effect of MPH treatment on reduced accident proneness might be better understood by the modulation via changed pain perception. Therefore the present findings could be seen as a further piece of the puzzle to fully understand the pathophysiology of ADHD.

## Limitations

Firstly, pain perception was not investigated using an experimental paradigm. The present data result from subjective questionnaires in which parents remembered painful events of their children and had to estimate the perceived severity of their children’s pain in the last 3 months. Also older children and adolescents were asked to estimate their own pain perception. Analyses of both datasets together as well as separately showed reduced pain perception in children with ADHD without MPH medication in comparison to both, HC and children with MPH medication. However, it will be advantageous to conduct further research in order to add more depth to the question of whether ADHD and MPH are associated with altered pain perception in children and adolescents. In addition imaging studies analyzing neural correlates of theses alterations might provide further insights into the etiopathophysiological mechanisms underlying abnormal pain perception in ADHD and the mechanisms of action of MPH on normalizing this abnormal pain perception in ADHD. Moreover, although analyses with the SDQ item “hyperactivity” and the “ADHD diagnosis question” were shown to be highly correlated, we could not entirely control for the diagnostic validity. Finally common and distinct effects of frequent comorbidities in ADHD as well as differences in age and diagnosis systems have to be considered i.e. when designing future research in this field.

## Conclusions

The present study found reports of reduced pain perception in children and adolescents with ADHD that seem to be normalized by MPH. If the suggested association between dopaminergic and potentially opioid deficits in the brain and altered pain perception could be shown experimentally in children and adolescents with ADHD, a further important step in the investigation of the pathophysiology of ADHD would be reached. The role of MPH in this assumed interplay might be to regulate and thus to balance dopaminergic and potentially opioid deficits which in turn might lead to an enhancement of pain perception up to the level of HC. Finally, not only in the context of MPH treatment, but also of behavioral treatment programs for ADHD, attention to reduced pain perception might help to further minimize the increased risk for injuries and accidents in those children with ADHD not treated with MPH.

## Abbreviations

ADHD: attention deficit hyperactivity disorder
ASD: autism spectrum disorders
CD: conduct disorders
MPH: methylphenidate
KiGGS: German health interview and examination survey for children and adolescents
MFNU: motor function neurological assessment
RKI: Robert Koch Institute
